# Combined analysis of the effects of hypoxia and oxidative stress on DNA methylation and the transcriptome in HTR‐8/SVneo trophoblast cells

**DOI:** 10.1111/jcmm.18469

**Published:** 2024-06-20

**Authors:** Xinjing Yan, Yang Fang, Yujie Yuan, Yangnan Ding, Haiyang Yu, Yina Li, Qianqian Shi, Yongrui Gao, Xinyuan Zhou, Dongxin Zhang, Enwu Yuan, Hongwei Zhou, Xin Zhao, Linlin Zhang

**Affiliations:** ^1^ Department of Laboratory Medicine Third Affiliated Hospital of Zhengzhou University Zhengzhou China; ^2^ Zhengzhou Key Laboratory for In Vitro Diagnosis of Hypertensive Disorders of Pregnancy Zhengzhou China; ^3^ Judicial Appraisal Institution Third Affiliated Hospital of Zhengzhou University Zhengzhou China; ^4^ Department of Obstetrics and Gynecology The Third Affiliated Hospital of Zhengzhou University Zhengzhou China; ^5^ Microbiome Medicine Center, Department of Laboratory Medicine Zhujiang Hospital, Southern Medical University Guangzhou China; ^6^ Tianjian Advanced Biomedical Laboratory Zhengzhou China

**Keywords:** DNA methylation, HTR‐8/SVneo trophoblast cells, hypoxia, oxidative stress, transcriptome

## Abstract

The alterations in DNA methylation and transcriptome in trophoblast cells under conditions of low oxygen and oxidative stress have major implications for pregnancy‐related disorders. However, the exact mechanism is still not fully understood. In this study, we established models of hypoxia (H group) and oxidative stress (HR group) using HTR‐8/SVneo trophoblast cells and performed combined analysis of genome‐wide DNA methylation changes using reduced representation bisulphite sequencing and transcriptome expression changes using RNA sequencing. Our findings revealed that the H group exhibited a higher number of differentially methylated genes and differentially expressed genes than the HR group. In the H group, only 0.90% of all differentially expressed genes displayed simultaneous changes in DNA methylation and transcriptome expression. After the threshold was expanded, this number increased to 6.29% in the HR group. Notably, both the H group and HR group exhibited concurrent alterations in DNA methylation and transcriptome expression within Axon guidance and MAPK signalling pathway. Among the top 25 differentially methylated KEGG pathways in the promoter region, 11 pathways were commonly enriched in H group and HR group, accounting for 44.00%. Among the top 25 KEGG pathways in transcriptome with significant differences between the H group and HR group, 10 pathways were consistent, accounting for 40.00%. By integrating our previous data on DNA methylation from preeclamptic placental tissues, we identified that the *ANKRD37* and *PFKFB3* genes may contribute to the pathogenesis of preeclampsia through DNA methylation‐mediated transcriptome expression under hypoxic conditions.

## 1 INTRODUCTION

The formation and physiological mechanisms of the placenta are crucial for normal foetal development.^1^ Throughout pregnancy, the DNA methylation and transcriptome levels of the placenta undergo dynamic changes. In early pregnancy, the placenta exhibits high transcriptional activity, with typical pathway enrichment and gene expression.^2^, ^3^ As gestational age increases, the overall methylation level of the placenta also alters.^4^, ^5^, ^6^, ^7^ Various factors, both exogenous (such as environmental pollution, drug exposure and social psychology) and endogenous (such as maternal physiological and metabolic conditions), can influence the DNA methylation status of the placenta.^8^, ^9^ These changes in DNA methylation can impact placental function^10^, ^11^ and lead to the release of inflammatory factors into the maternal circulation, resulting in a range of pregnancy complications.^12^

In recent years, there has been growing interest in understanding the role of oxygen concentration changes in the physiological and pathological mechanisms of placental function during the first and second trimesters.^13^, ^14^ The transition from hypoxia to oxidative stress is associated with major morphological and physiological changes in the placenta, including villus regression, placental formation and foetal membrane development,^15^ Hormone secretion, transcription factor expression, protein processing and transport all contribute to this process. DNA methyltransferases (DNMTs) are crucial enzymes involved in methylation changes that affect trophoblast cell function under conditions of hypoxia and oxidative stress. These enzymes are also implicated in the development of preeclampsia, an important pregnancy disorder.^16^, ^17^, ^18^ However, our understanding of specific alterations in DNA methylation patterns and transcriptome expression as well as regulatory factors and signalling pathways occurring during placental oxygen transition remains limited.

In this study, we utilized an in vitro trophoblast cell line (HTR‐8/SVneo) to establish hypoxic and oxidative stress models. We compared the differentially expressed genes and signalling pathways in DNA methylation and transcriptome expression between these two models. Our aim was to enhance our understanding of the regulatory mechanisms and specific signalling pathways involved in the impact of hypoxia and oxidative stress on trophoblast cell function during pregnancy. Additionally, we integrated the sequencing results with DNA methylation data from preeclampsia (PE) to provide valuable insights into abnormal placental development and pregnancy‐related disorders.

## 2 MATERIALS AND METHODS

### 2.1 Cell culture and treatment

The HTR‐8/SVneo trophoblast cells utilized in this study were sourced from the American Type Culture Collection. The cells were cultured in RPMI 1640 medium (Solarbio, 31,800) supplemented with 10% foetal bovine serum (Genial, G24‐70500), penicillin (100 U/mL) and streptomycin (100 μg/mL) antibiotic. The control group cells were maintained under 20% oxygen conditions. The hypoxic group cells (H group) were exposed to 1% oxygen concentration for various durations (12, 24, 48, 72 and 96 h) within a humidified hypoxia workstation (Invivo2 400). Cells in oxidative stress group (hypoxia‐reoxygenation, HR group) were treated with hypoxia and reoxygenation as the follows: the 12‐h treatment time was treated in 1% oxygen concentration for 6 h and then placed in 20% oxygen concentration for 6 h. The 24‐h treatment time was treated in 1% oxygen concentration for 12 h and then put in 20% oxygen concentration for 12 h. Taking 24‐h treatment time as a cycle, the cells were treated for 48 h (2 cycles), 72 h (3 cycles) and 96 h (4 cycles). Based on the results of the experiments on cell function, we selected cells with good cell viability at 48 h for sequencing analysis and validation experiments. Protein and RNA samples were collected within 1 min of completing the treatments.

### 2.2 Quantitative real‐time polymerase chain reaction (qRT‐PCR) experiments

The TRIzon Total RNA Extraction Kit (CW0580S) was used to extract RNA samples. The extracted RNA was then reverse‐transcribed into cDNA using the ReverTra Ace qPCR RT Master Mix (FSQ‐201) reagent. qRT‐PCR was performed using the UltraSYBR Mixture (CW0957M) reagent for polymerase chain reaction amplification. The relative expression levels of all target genes were calculated using the $2^{-\Delta \Delta c_{t}}$ analytical method, with *GAPDH* serving as the endogenous control gene. Those experiments were performed with three biological replicates with HTR‐8/SVneo cells. The qRT‐PCR primer sequences of the endogenous control and target genes are shown in Table 1.

**TABLE 1:** Primer sequences for qRT‐PCR experiments.

Name		Sequence
*DNMT1*	Forward	AAGAGCCAAATCGGATGAGT
	Reverse	AAGCGGTCTAGCAACTCGTT
*DNMT3a*	Forward	GGTGTGGCTTTAGGAGCAGT
	Reverse	CTACAGGCAGGTCAGTGAGC
*FN1*	Forward	GAGAATAAGCTGTACCATCGCAA
	Reverse	CGACCACATAGGAAGTCCCAG
*TGFBI*	Forward	CACTCTCAAACCTTTACGAGACC
	Reverse	CGTTGCTAGGGGCGAAGATG
*TFRC*	Forward	ACCATTGTCATATACCCGGTTCA
	Reverse	CAATAGCCCAAGTAGCCAATCAT
*IL32*	Forward	TGGCGGCTTATTATGAGGAGC
	Reverse	CTCGGCACCGTAATCCATCTC
*FOS*	Forward	CACTCCAAGCGGAGACAGAC
	Reverse	AGGTCATCAGGGATCTTGCAG
*PFKFB3*	Forward	GCGTCCCCACAAAAGTGTTC
	Reverse	TGGAGGTTGTGCTCGTTCTC
*ANKRD37*	Forward	AGAAGCTCCACTACACAAGG
	Reverse	AATTTTGGGCATCACTGGCTAC
*GAPDH*	Forward	AGAACGGGAAGCTTGTCATC
	Reverse	CATCGCCCCACTTGATTTTG

*Note*: All sequences are written in 5′–3′ direction.

### 2.3 Western blot (WB) experiments

Electrophoretic gels for WB experiments were configured with the Omni‐Easy™ one‐step PAGE Gel Rapid Preparation Kit (Yazyme, PG212). Protein samples were separated by electrophoresis and transferred to the 0.45‐micron polyvinylidene fluoride (PVDF) membranes and incubated overnight at 4°C with rabbit anti‐human monoclonal antibodies against DNMT1 (ab188453) and DNMT3a (ab188470) and rabbit anti‐human β‐actin antibody (81115‐1‐RR). The following day, the membranes were incubated with goat anti‐rabbit IgG (IRDye 680RD) fluorescent secondary antibody for 1 h. The grey values of the protein bands were analysed using Image J software after scanning and analysis of the gel imaging system. The relative expression of target proteins was analysed by using *β‐actin* as the endogenous control gene. These experiments were performed with three biological replicates with HTR‐8/SVneo cells.

### 2.4 Cell viability, migration and invasion assays

According to the instructions, the Cell Counting Kit‐8 (CCK‐8) (MA0218) cell proliferation and toxicity assay kit was used to assess changes in cell viability after hypoxia and oxidative stress. The cells in the six‐well plate were scratched in longitudinal lines, and then the wound was photographed under a microscope. The area of the wound in the images was calculated using Image J software. The wound healing percentage was determined using the formula (A0 – A24/48)/A0 × 100%, where A0/24/48 represents the scratch area at 0 h, 24 h and 48 h. Matrigel (Corning 356,234) was diluted at a ratio of 1:8 and applied to the bottom of a chamber. After the upper chamber was hydrated, a 200 μL cell suspension in serum‐free medium was added to the upper chamber, while 600 μL of 10% FBS medium was added to the lower chamber. The cells were fixed with 4% paraformaldehyde and stained with crystal violet, and seven fields per chamber were photographed to calculate the mean cell number. These experiments were performed with three biological replicates with HTR‐8/SVneo cells.

### 2.5 Cellular ROS fluorescence intensity and SOD content

According to the reagent instructions, the cells were subjected to treatment using the reactive oxygen species (ROS) detection kit (CA1410). Subsequently, five photos per well were captured using an inverted phase contrast fluorescence microscope for average fluorescence intensity analysis using Image J software. Total superoxide dismutase (SOD) activity was measured through colorimetry utilizing a total superoxide dismutase detection kit (S0103). The SOD content per unit protein concentration of each sample was determined. These experiments were performed with three biological replicates with HTR‐8/SVneo cells.

### 2.6 DNA methylation analysis

The con, H and HR groups were set up with three biological replicates each, resulting in a total of nine samples were detected by Reduced representation bisulphite sequencing (RRBS) technology in Shenzhen Aisi Gene Technology Co., Ltd. DNA samples were treated with bisulphite to construct DNA libraries. After qualified library quality inspection, different libraries were pooled according to the requirements of effective concentration and target amount of data, and then Illumina HiSeq sequencing was performed. Bsmap software was used to analyse the alignment of methylation data to the reference genome, and the methylation sites were detected. The methylation levels of the sites were calculated using the formula: methylation level = mC/(mC + umC). The differentially methylated regions (DMRs) were obtained by metilene software and multiple test correction (thresholds: |Diff| ≥ 0.1 and *p*_*adjust*_ <0.05). Based on the DMRs results, Gene Ontology (GO, https://www.geneontology.org/, thresholds: Ovserved>2 FoldChange>2 and *p* < 0.05) functional enrichment analysis and Kyoto Encyclopedia of Genes and Genomes (KEGG, https://www.genome.jp/kegg/, thresholds: Ovserved>2 FoldChange>2 and *p* < 0.05) pathway enrichment analysis were performed for the genomes overlapped with DMRs and their upstream and downstream and other regions.

Targeted bisulphite sequencing (TBSeq panel) was used to validate the DMRs. BS‐PCR primers were designed using the online MethPrimer software, and the primer sequences of the target genes are shown in Table 2. DNA samples were amplified using Acegen Targeted Methyl Panel Kit, transformed with bisulphite and purified. DNA libraries were constructed using Acegen DNA Library Prep Kit and Agencourt® AMPure® XP (nucleic acid Purification kit). The NovaSeq Reagent Kit (PE150) was used to sequence the library after quality inspection. The methylation level of CG base on each target sequence of each sample was calculated, and the methylation level was calculated according to the following formula: methylation level of C site = number of reads supporting methylation/(number of reads supporting unmethylation), and the methylation level of each site in each sample was counted.

**TABLE 2:** Primer sequences for BS‐PCR experiments.

Name		Sequence
*ANKRD37*	Forward	YGGGGAGGTTTTTAGTTYGGAGGGG
	Reverse	AATAAACAAACRACTACTTACAAAAATCC
*PFKFB3*	Forward	GGGYGGYGGTTTTTTATTAAAAGTAATTTAG
	Reverse	CTACCTCCCCACTTTTTAATACATAC
*PTP4A2P1*	Forward	GTAAAGATTATATGTTGATTATTAAATAGGTTATTTG
	Reverse	TTCCTACTACYGCYGCTATCCTATAAC
*ANKMY1*	Forward	AGGAGGYGTYGTTAATAGYGGGTTTTAG
	Reverse	ATCTCCCTACTATCCRACRCRAACTC
*MIR1271_ARL10*	Forward	GGAAYGGTTTTGTTGGTGTYGGGAG
	Reverse	AAATACTCCCRAACCRCRCCCTCCAAA
*IL32*	Forward	TGGCGGCTTATTATGAGGAGC
	Reverse	CTCGGCACCGTAATCCATCTC
*FOS*	Forward	CACTCCAAGCGGAGACAGAC
	Reverse	AGGTCATCAGGGATCTTGCAG
*PFKFB3*	Forward	GCGTCCCCACAAAAGTGTTC
	Reverse	TGGAGGTTGTGCTCGTTCTC
*ANKRD37*	Forward	AGAAGCTCCACTACACAAGG
	Reverse	AATTTTGGGCATCACTGGCTAC
*GAPDH*	Forward	AGAACGGGAAGCTTGTCATC
	Reverse	CATCGCCCCACTTGATTTTG

*Note*: All sequences are written in 5′–3′ direction.

### 2.7 Transcriptome sequencing analysis

Three biological repeated experiments with HTR‐8/SVneo cells were set up in con, H and HR groups, and a total of nine cell RNA samples were detected by RNA sequencing (RNA‐Seq) technology by Shanghai Meiji Biomedical Technology Co., Ltd. mRNA was isolated from total RNA using magnetic beads with oligo dT, which facilitated A‐T base pairing and poly‐A selection. The isolated mRNA was then subjected to fragmentation using fragmentation buffer, resulting in fragments of approximately 300 bp. Reverse transcriptase was used to synthesize cDNAs from these fragments. Adaptors were ligated to the cDNAs, and the resulting libraries were sequenced on an Illumina platform. The data were analysed on the online platform of Majorbio Cloud Platform (https://www.majorbio.com). For differentially expressed genes (DEGs), these with |log2FC| ≥ 1.00 and *p*_*adjust*_ < 0.05 were considered to have significant differences. KEGG analysis was performed using *p* < 0.05 to identify significant differences.

### 2.8 Statistical analysis

GraphPad Prism 9 software was utilized for statistical analysis. Normally distributed data were analysed using a t test or one‐way analysis of variance (ANOVA). For data that were not normally distributed, the nonparametric Mann–Whitney test was employed. The results with a *p* < 0.05 were deemed statistically significant.

## 3 RESULTS

### 3.1 Hypoxia and oxidative stress affect trophoblast function and DNA methylation levels

**FIGURE 1:**
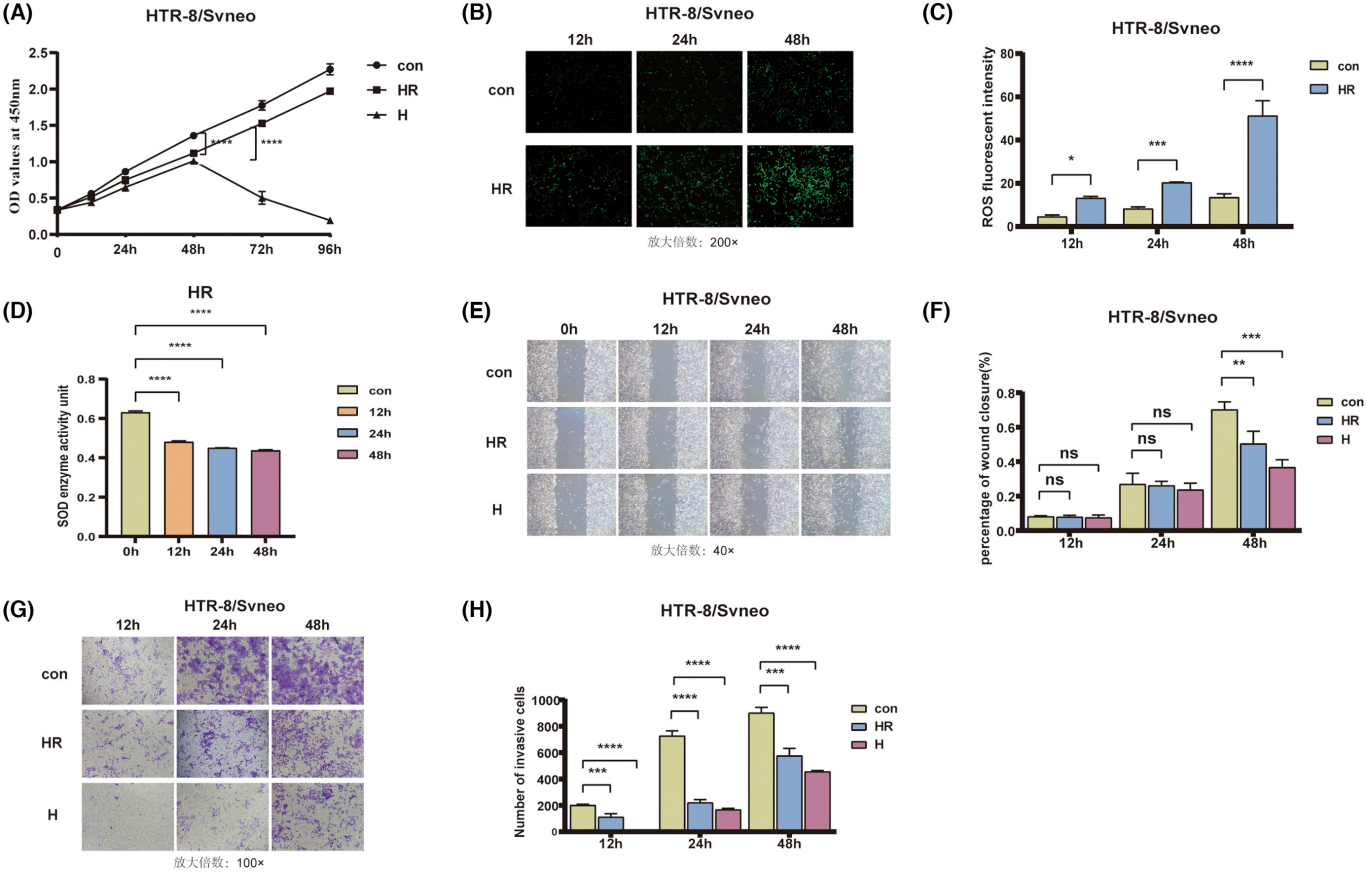
Establishment of the hypoxic and oxidative stress models. (A) Decreased cell viability following exposure to hypoxia and oxidative stress. (B–D) increased reactive oxygen species (ROS) content and decreased superoxide dismutase (SOD) content after oxidative stress. (E–H) The ability of cells to migrate and invade was reduced under hypoxic and oxidative stress conditions. *p < 0.05, **p < 0.01, ***p < 0.001, ****p < 0.0001. con: control group; H: hypoxic group; HR: hypoxia reoxygenation, as the oxidative stress group.

At an oxygen concentration of 1%,^19^ the cell viability of both the H group and the HR group decreased with the extension of treatment time (Figure 1A). Notably, the cell viability of the H group significantly decreased at 72 h, rendering these cells unsuitable for further analysis. Consequently, cells with good viability that were treated for 48 h were selected for subsequent experiments. Compared to the control cells, the cells in the HR group exhibited a gradual increase in ROS accumulation (Figure 1B,C), accompanied by a gradual decrease in SOD content (Figure 1D). After 48 h of treatment, the cell's ability to migrate and invade was significantly reduced (Figure 1E–H). These experiments indicated that the cell model of hypoxia and oxidative stress was successfully established. Additionally, the protein and RNA expression levels of DNMT1 and DNMT3a were decreased (Figure S1A–F), indicating that the DNA methylation level of trophoblast cells was altered.

### 3.2 Changes in DNA methylation in trophoblasts following hypoxia and oxidative stress

**FIGURE 2:**
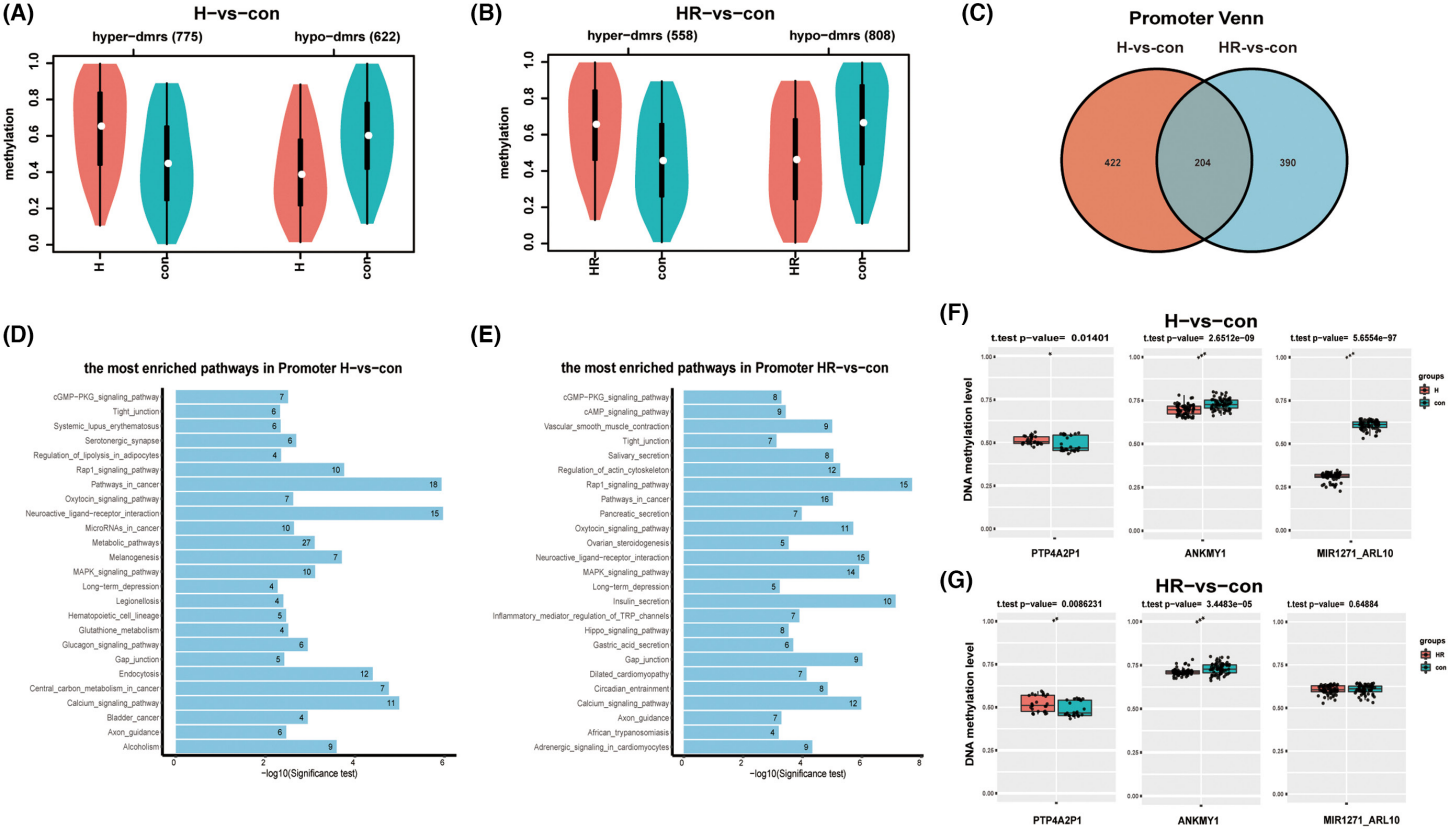
Analysis and validation of reduced representation bisulphite sequencing (RRBS). (A, B) Changes in methylation sites following hypoxia and oxidative stress (|Diff| ≥ 0.1, *p*_*adjust*_ < 0.05). (C) Majority of the differentially expressed genes between H group and HR group appeared were located in the Promoter region, with 204 genes overlapping. (D, E) There were 11 overlapping pathways in the top 25 KEGG pathway enrichment in Promoter region between H group and HR group (Ovserved>2, FoldChange >2 and *p* < 0.05). (F, G) TBS validation demonstrated hypermethylation of *PTP4A2P1* and hypomethylation of *ANKMY1* and *MIR1271‐ARL10* in the H group. In the HR group, *PTP4A2P1* was hypermethylated, *ANKMY1* was hypomethylated and *MIR1271‐ARL10* showed no significance. *p < 0.05, **p < 0.01, ***p < 0.001. con: control group; H: hypoxic group; HR: hypoxia reoxygenation, as the oxidative stress group.

The RRBS results showed, compared with the control group, there were 775 up‐regulated DMRs and 622 down‐regulated DMRs in the H group (thresholds: |Diff| ≥ 0.1 and *p*_*adjust*_ <0.05) (Figure 2A), and 558 up‐regulated DMRs and 808 down‐regulated DMRs in the HR group (thresholds: |Diff| ≥ 0.1 and *p*_*adjust*_ <0.05) (Figure 2B). Among these DMRs, 204 DMRs overlapped in the promoter region of H group and HR group, accounting for 32.59% of all DMRs in the promoter region of the H group and 34.34% of all DMRs in the promoter region of the HR group (Figure 2C). In the GO functional annotation analysis of the promoter region, 16 of the top 25 differentially functional regions overlapped in H group and HR group (thresholds: Observed >2, FoldChange >2, *p* < 0.05) (Figure S2A,B), accounting for 64%. Among the top 25 KEGG differential pathways, 11 pathways were overlapped in the H group and HR group (thresholds: Observed >2, FoldChange >2), (*p* < 0.05), accounting for 44.00%. Additionally, among the remaining 14 non‐overlapping pathways, DNA methylation changes in the H group were primarily concentrated in central carbon metabolism in cancer, endocytosis and melanogenesis, while changes in the HR group were mainly associated with regulation of inflammatory mediators and secretion of saliva, pancreas and gastric acid (Figure 2D,E).

The TBS sequencing experiments validated three DMRs with significant differential methylation in the promoter region of the H group and the HR group: *PTP4A2P1*, *ANKMY1* and *MIR1271‐ARL10*. Except for the M*IR1271‐ARL10* gene in the HR group, which showed no significant difference in promoter methylation, the expression of the remaining genes in both the H and HR groups was consistent with the results of RRBS (Figure 2F,G).

### 3.3 Altered trophoblast transcriptome expression following hypoxia and oxidative stress

**FIGURE 3:**
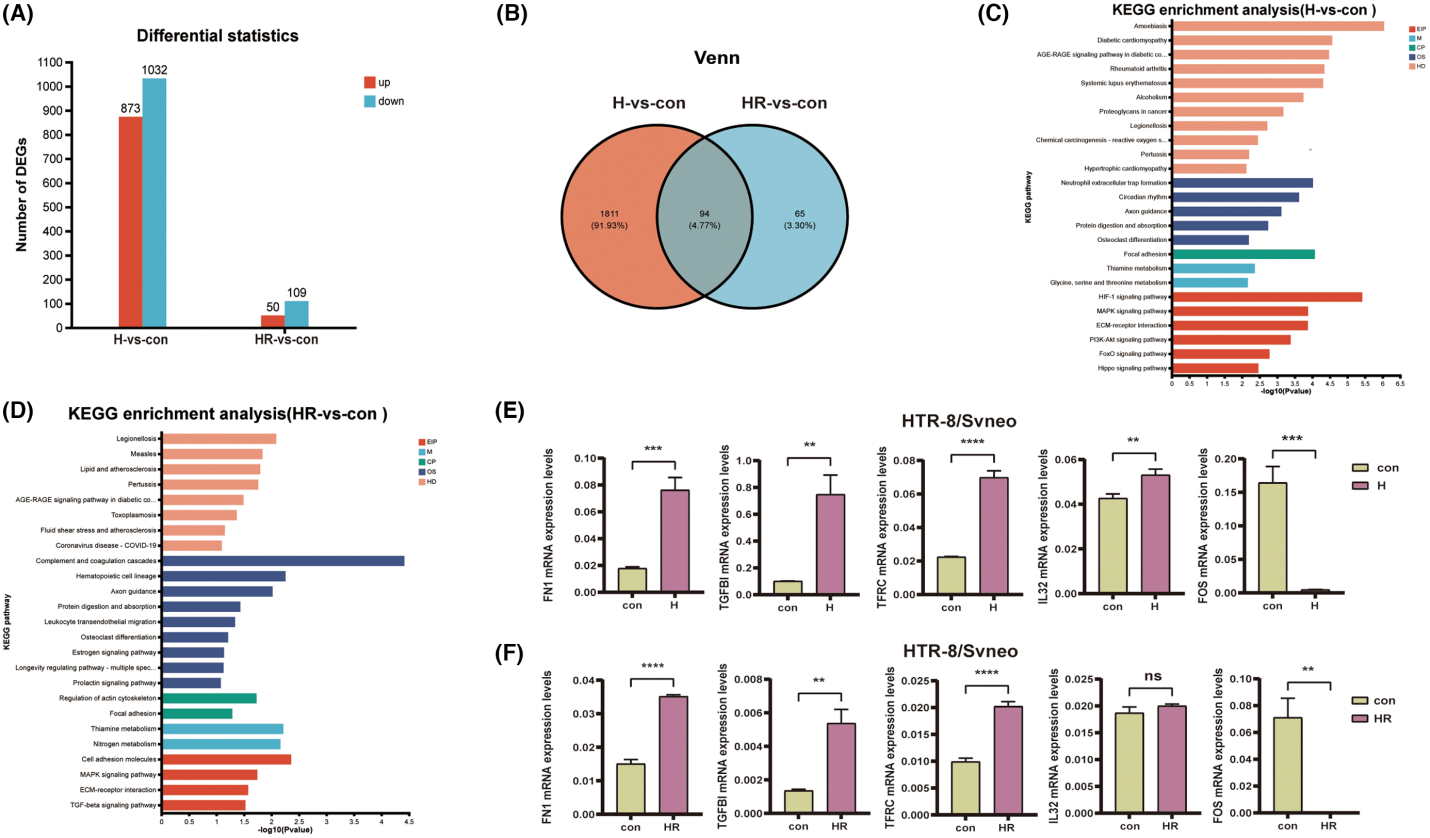
Analysis and validation of RNA sequencing. (A) Differential expression of genes (DEGs) following hypoxia and oxidative stress (|log2FC| ≥ 1.00, *p*_*adjust*_ < 0.05). (B) There were 94 overlapping DEGs between H group and HR group. (C, D) 10 overlapping pathways were identified among the top 25 pathways in KEGG pathway enrichment (*p* < 0.05). (E, F) qRT‐PCR results showed increased expression of *FN1*, *TGFBI*, *TFRC*, *IL32* and *FOS* in the H group. The expression of the remaining four genes was increased in the HR group, except for *IL32*. **p < 0.01, ***p < 0.001, ****p < 0.0001. con: control group; H: hypoxic group; HR: hypoxia reoxygenation, as the oxidative stress group.

RNA‐seq results showed, compared with control group, that the H group has 873 DEGs increased expression and 1032 DEGs expression is reduced, the HR group has 50 DEGs higher expression and reduce 109 DEGs expression (thresholds: |log2FC| ≥ 1.00, *p*_*adjust*_ < 0.05) (Figure 3A). There were 94 overlap DEGs between H group and HR group, which accounting for 4.93% of all DEGs in the H group and 59.11% of all DEGs in the HR group (Figure 3B). In the GO functional annotation analysis, 22 of the top 25 differentially functional regions overlapped in H group and HR group (Figure S3A,B), accounting for 88%. In terms of KEGG pathway enrichment, 10 of the 25 pathways with significant differences between the H group and HR group were overlapping (*p* < 0.05), accounting for 40.00% (Figure 3C,D). In addition, among the remaining 15 non‐overlapping pathways, the H group showed significant differences in the HIF‐1 signalling pathway, some immune system diseases and PI3K‐Akt signalling pathway (Figure 3C), and the HR group showed significant differences in some blood system and TGF‐β signalling pathway (Figure 3D).

The qRT‐PCR experiments were used to verify the top 10 genes in the common DEGs of the H group and HR group, and their gene expression levels were more than 25: *FN1*, *TGFBI*, *TFRC*, *IL32* and *FOS*. We found that the expression of the five DEGs in H group was consistent with the sequencing results (Figure 3E). Except for *IL32*, the expression of the remaining four genes in the HR group was consistent with the sequencing results (Figure 3F). qRT‐PCR verification results increased the confidence of the RNA sequencing results.

### 3.4 Combined analysis of DNA methylation sequencing and transcriptome sequencing results

**FIGURE 4:**
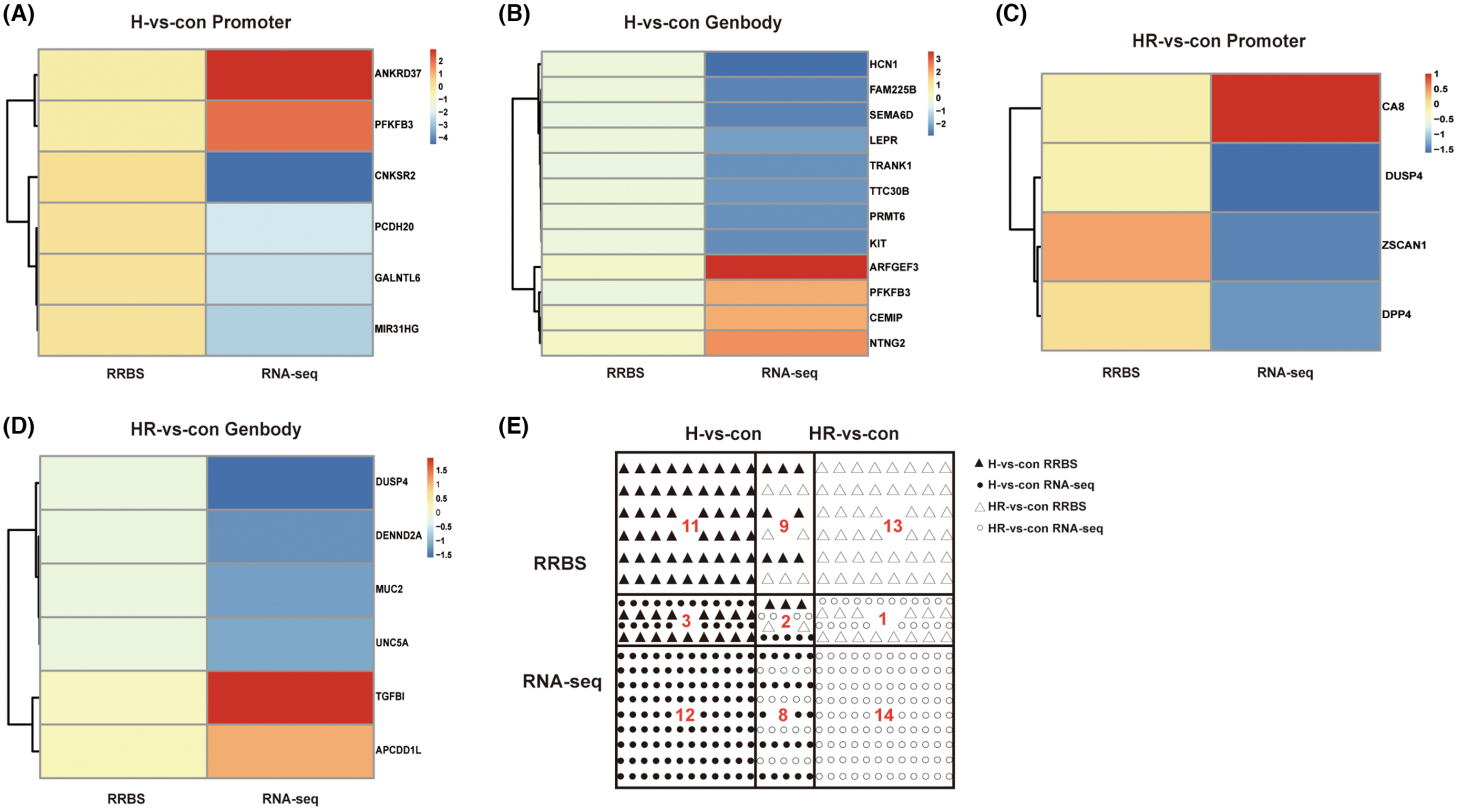
Impact of altered DNA methylation on transcriptome expression. (A, B) Overlapping differentially expressed genes (DEGs) and differentially methylated regions (DMRs) in the Promoter and Genbody regions of the H group (|log2FC| ≥ 2.00, |Diff| ≥ 0.1, *p* < 0.05). (C, D) Overlapping DEGs and DMRs in the Promoter region and Genbody region of the HR group (|log2FC| ≥ 1.00, |Diff| ≥ 0.05, *p* < 0.05). (E) Combined analysis of 25 KEGG signalling pathways from promoter region DNA methylation sequencing and 25 KEGG signalling pathways from transcriptome sequencing in the H group and HR group. RRBS: Reduced Representation Bisulphite Sequencing, used to detect DNA methylation levels; RNA‐seq: Transcriptome sequencing, used to detect gene expression levels. con: control group; H: hypoxic group; HR: hypoxia reoxygenation, as the oxidative stress group. The presence of multiple icons within the figures represented the intersection of corresponding datasets.

Gene DNA methylation changes can lead to alterations in transcriptome expression. In the paired analysis of DEGs and DMRs in H group, 17 genes overlapped (thresholds: |log2FC| ≥ 2.00, |Diff| ≥ 0.1, *p* < 0.05), accounting for 0.90% of the transcriptome DEGs. Among these genes, 12 were located in the gene body region and 6 were located in the promoter region (Figure 4A,B). Notably, the *PFKFB3* gene and the *ANKRD37* gene exhibited significant differences. The combined analysis showed that DEGs and DMRs in HR group did not overlap. After the threshold was expanded (enlarged thresholds for |log2FC| ≥ 1.00, |Diff| ≥ 0.05, *p* < 0.05), there were 10 genes that overlapped, accounting for 6.29% of the transcriptome DEGs. Among these genes, six were distributed in the gene body region and four were distributed in the promoter region. The *CA8* gene showed a significant difference among these overlapping genes (Figure 4C,D).

In the joint analysis of KEGG pathways between DNA methylation sequencing and transcriptome sequencing in the H group, we identified five overlapping pathways, namely MAPK signalling pathway, alcoholism, axon guidance, legionellosis and systemic lupus erythematosus. In the HR group, the joint analysis of KEGG pathways between DNA methylation sequencing and transcriptome sequencing revealed three overlapping pathways: MAPK signalling pathway, Axon guidance and Regulation of actin cytoskeleton (Figure 4E). Interestingly, DNA methylation and transcriptome expression of the MAPK signalling pathway and Axon guidance were simultaneously changed in the H and HR groups. This suggests that both hypoxia and oxidative stress can induce DNA methylation changes in the MAPK signalling pathway and Axon guidance leading to alterations in transcriptome expression.

### 3.5 Trophoblast RNA‐seq data, RRBS methylation data and PE placental tissue DNA methylation chip data were jointly analysed

**FIGURE 5:**
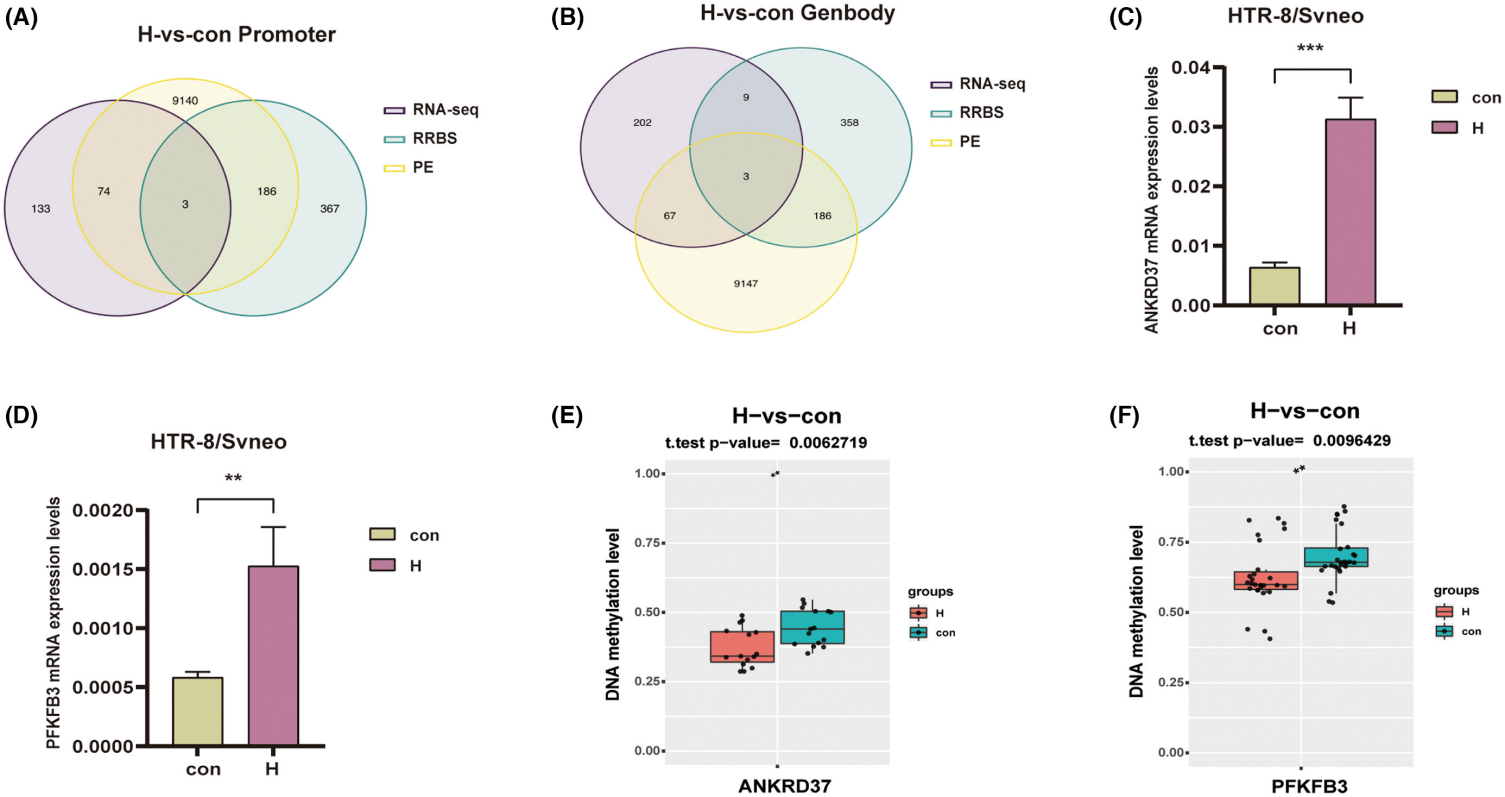
RRBS methylation data, RNA‐seq data and DNA methylation chip data of PE placenta tissue were jointly analysed in the H group. (A) *ANKRD37*, *PFKFB3* and *LINC01291* were hypomethylated and highly expressed in the Promoter region of H group and showed hypomethylation in the DNA methylation microarray data of PE tissues. (B) *KCNJ8*, *HCN1* and *TRANK1* were hypomethylated and down‐regulated in the Genbody region of H group and showed hypomethylation in the DNA methylation microarray data of PE tissues. (C, D) The mRNA expression levels of *ANKRD37* and *PFKFB3* were increased in the H group. (E, F) TBS experiments revealed reduced levels of ANKRD37 and PFKFB3 DNA methylation in H group. **p < 0.01; ***p < 0.001. con: control group; H: hypoxic group; RRBS: Reduced representation bisulphite sequencing; RNA‐seq: RNA sequencing; PE: Preeclampsia.

The Illumina Infinium HumanMethylation 850 K Bead Chip was utilized to investigate DNA methylation differences in placentas from women with PE, preterm birth and normal delivery.^20^ To explore the relationship among cellular hypoxia, oxidative stress‐related DNA methylation of genes and preeclampsia, we combined RNA‐seq data, RRBS methylation data and PE methylation chip data and found that there were 3 genes in the promoter region of H group: *ANKRD37*, *PFKFB3* and *LINC01291* (Figure 5A), and there were 3 genes in the genebody region of H group: *KCNJ8*, *HCN1* and *TRANK1* (Figure 5B). No overlapping genes were found in the joint analysis of the HR group. TBS assay and qRT‐PCR verified that *PFKFB3* and *ANKRD37* decreased DNA methylation (Figure 5E,F) and increased transcriptome expression levels in HTR‐8/SVneo cells after exposure to hypoxia (Figure 5C,D).

## 4 DISCUSSION

After treatment with the established regimen, HTR‐8/SVneo cells showed decreased viability, increased ROS accumulation and decreased SOD content and were characterized by impaired physiological mechanisms. These results were further confirmed by decreased cell migration and invasion, which were very similar to the characteristics of preeclamptic placental tissue. Interestingly, in a comparison of the hypoxia model to the oxidative stress model at the same treatment duration, the hypoxia model demonstrated more pronounced alterations in cell function and expression of DNMTs and differentially expressed genes. Additionally, when integrating the PE data, we identified two gene sets in the H group, while no gene set was formed in the HR group. This finding suggests that hypoxia exerts a greater impact on trophoblast function and gene expression.

The identified DMRs (1397 in the H group and 1366 in the HR group) and DEGs (1905 in the H group and 159 in the HR group) indicate extensive changes in DNA methylation and transcriptome expression during the transition from hypoxia to oxidative stress in trophoblast cells. Combined analysis of DNA methylation and transcriptome data revealed that 17 genes overlapped in the H group, accounting for 0.90% of transcriptome DEGs. HR group jointed analysis of DEGs and DMRs no overlap, after the threshold was expanded, there were 10 genes overlapped, accounting for 6.29% of the transcriptome DEGs. This finding suggests that DNA methylation has a limited impact on transcriptome changes in trophoblast cells stimulated by hypoxia and oxidative stress; other mechanisms may play a more important role. In the bioinformatics analysis, we found that DNA methylation and transcriptome expression of the Axon guidance and the MAPK signalling pathway were simultaneous altered in H group and HR group, suggesting that hypoxia and oxidative stress may affect trophoblast function by altering the DNA methylation level of the Axon guidance and the MAPK signalling pathway to affect transcriptome expression. Previous studies have indicated that the MAPK signalling pathway may regulate RNA m5C methylation levels and contribute to the pathogenesis of preeclampsia.^21^ Additionally, this pathway can regulate transcription factor activity, nuclear translocation, oxygen concentration,^22^, ^23^ TGF‐β1 and regulatory factors and participate in regulating trophoblast function, stress response, inflammatory response and other physiological/pathological processes. This pathway is closely associated with preeclampsia,^24^ recurrent spontaneous abortion,^25^ and other diseases. Previous studies have shown that the receptors of axon‐guiding factor neurociliin‐1 and neurociliin‐2 usually combine with vascular endothelial growth factor (VEGF) and placental growth factor (PlGF) to play a role in axon‐guiding, which can promote angiogenesis and cell migration in endothelial cells.^26^

GO functional annotation analysis revealed that the DMRs and DEGs in both the H group and the HR group exhibited similar functional changes. The common DMRs in H group and HR group were primarily associated with gene expression regulation, cell signal transduction, cell morphological changes and nutrient transmission. The functional changes of the common DEGs were mainly related to biological processes and biological regulation at the organelle level. These findings suggest that both hypoxia and oxidative stress may involve similar gene functions in the physiological and pathological mechanisms of placental development, as observed from both DNA methylation and transcriptome expression perspectives.

The effects of certain pathways on placental trophoblasts may persist from a hypoxic state to an oxidative stress state. In the KEGG pathway enrichment analysis of DNA methylation conducted in this study, 11 common differential pathways were identified between the H group and HR group. These pathways were involved in the physiological and pathological mechanisms of trophoblast differentiation, adhesion, signal transduction, cell contraction, secretion, migration, invasion, etc. The 10 pathways with common changes in the transcriptome of the H group and HR group were involved in trophoblast proliferation and differentiation, inflammatory response, extracellular matrix remodelling, cell migration, etc. Notably, the MAPK signalling pathway, ECM‐receptor interaction, Rap1 signalling pathway, cGMP‐PKG signalling pathway and oxytocin signalling pathway were affected by both hypoxia and oxidative stress. These findings align with a previous study on DNA methylation and transcript expression differences in placental chorionic villi during the first and second trimesters of pregnancy.^27^ These results suggest that hypoxia and oxidative stress primarily impact trophoblast function through these signalling pathways during early pregnancy. Yuen RK et al. found that hypoxia‐induced gene hypermethylation and transcriptomic expression changes were mainly co‐enriched in the phosphoprotein signalling pathway. However, due to methodological limitations, no DNA hypomethylation sites were found in trophoblasts induced by hypoxia in the article.^28^ In this study, DNA methylation changes in phosphoprotein binding function in the H group were found in GO functional annotation analysis, but no methylation changes in phosphoprotein‐related signalling pathways or transcript expression changes were found. The reason for the inconsistent results may be related to the different specimen types used.

The effects of hypoxia and oxidative stress on trophoblasts had different emphases. In the transcript analysis of this study, the H group showed significant enrichment in the HIF‐1 signalling pathway, PI3K‐Akt signalling pathway and immune system diseases. It has been observed that the hypoxic environment can activate the HIF‐1 signalling pathway to enhance the transcription of angiogenic factors (such as VEGF)^29^ and metabolic regulatory factors (such as GLUT1),^30^, ^31^ thereby promoting cell survival and adaptability.^32^ The PI3K‐Akt signalling pathway aids in cellular survival and adaptation to hypoxia by facilitating cell metabolism and antioxidant capacity.^33^, ^34^ Hypoxic conditions may alter the secretion pattern and expression levels of immunomodulatory factors in trophoblast cells, leading to increased production of specific cytokines and regulatory molecules such as IL‐6, IL‐8 and VEGF,^35^, ^36^ which help trophoblast cells adapt to hypoxia while regulating immune responses.^37^ In certain cases, these changes are associated with pregnancy complications such as preeclampsia and placental insufficiency.^38^, ^39^ We observed a significant enrichment of HR group transcripts primarily in the TGF‐β signalling pathway, which has been demonstrated to mitigate oxidative stress by upregulating activin A expression, maintaining placental function,^40^ and regulating various biological processes, including cell proliferation, differentiation and immune regulation. Furthermore, hypoxia‐induced DNA hypermethylation changes have been associated with delayed cell differentiation.^41^ Among the differentially expressed pathways identified in this study, the PI3K‐Akt signalling pathway has been implicated in regulating the development of the differentiated trophoblast giant cell phenotype.^42^ TGF‐β signalling has been shown to govern the differentiation program of extrachorionic trophoblasts.^43^

In this study, we observed that *ANKRD37* and *PFKFB3* exhibited hypomethylation and high expression in HTR‐8/SVneo cells after hypoxia, and they also showed low methylation in preeclampsia placental tissues. By reviewing the literature, we discovered that *ANKRD37* and *PFKFB3* were also identified in the PE placenta transcriptome database by Gong et al., with *ANKRD37* showing high expression in PE placental tissue (*p* < 0.05).^44^ *ANKRD37* is a novel target gene of HIF and is involved in the regulation of key transcri*p*tion factors such as NF‐κB and TP53, suggesting its potential role in the hypoxia response.^45^ Increased expression of *ANKRD37* has been observed in preeclamptic placentas and may inhibit trophoblast cell migration and invasion through the NF‐κB pathway.^46^ Researchers propose that *ANKRD37* could serve as a novel marker for PE.^47^ *PFKFB3*, in contrast, is an enzyme involved in glucose metabolism. In our study, *PFKFB3* was enriched in the transcripts of the HIF‐1 signalling pathway in the H group and the fructose and mannose metabolism pathway in the HR group. Previous studies have shown that *PFKFB3* can promote placental angiogenesis, enhance placental function,^48^ regulate the LPS‐induced inflammatory response through the NF‐κB pathway in HTR‐8/SVneo cells, and inhibit cell invasion and migration.^49^ Therefore, hypoxia‐induced DNA methylation changes in the promoter regions of *ANKRD37* and *PFKFB3* are likely to alter their gene expression levels, potentially contributing to the pathogenesis of PE.

In this study, we jointly analysed DNA methylation and transcriptome changes in the model of hypoxia and oxidative stress in extravchorionic trophoblasts to provide insights into the specific signalling pathways and mechanisms of hypoxia and oxidative stress during pregnancy in regulating the function of trophoblast lines. This is the first study to comprehensively investigate the association between DNA methylation changes under hypoxic conditions in extravillous trophoblasts during early pregnancy and preeclampsia. However, there are certain limitations to our study. In vitro models cannot fully capture the complexity of mechanisms occurring during pregnancy. In this study, the human trophoblast cell line HTR‐8/SVneo was used as an in vitro model, but primary trophoblast cells or in vivo experimental verification was not used, which will be verified in subsequent experimental studies.

## AUTHOR CONTRIBUTIONS

**Xinjing Yan:** Data curation (equal); methodology (equal); validation (equal); writing – original draft (equal). **Yang Fang:** Data curation (equal); formal analysis (equal); software (equal); visualization (equal). **Yujie Yuan:** Validation (equal). **Yangnan Ding:** Formal analysis (equal). **Haiyang Yu:** Methodology (equal). **Yina Li:** Validation (equal). **Qianqian Shi:** Methodology (equal); validation (equal). **Yongrui Gao:** Investigation (equal). **Xinyuan Zhou:** Investigation (equal). **Dongxin Zhang:** Data curation (equal). **Enwu Yuan:** Project administration (equal); resources (equal). **Hongwei Zhou:** Methodology (equal). **Xin Zhao:** Supervision (equal). **Linlin Zhang:** Conceptualization (equal); methodology (lead); supervision (lead); writing – review and editing (lead).

## FUNDING INFORMATION

Henan province medical science research plan province department of construction of key projects (SBGJ202302085).

## CONFLICT OF INTEREST STATEMENT

The authors declare that they have no competing interests.

## Supporting information


Figure S1.

Figure S2.

Figure S3.

## Data Availability

The datasets used and/or analysed during the current study are available from the corresponding author on reasonable request.
