# Supplementary figures and images for: Combined analysis of the effects of hypoxia and oxidative stress on DNA methylation and the transcriptome in HTR‐8/SVneo trophoblast cells
Source: J Cell Mol Med. 2024 Jun 20;28(12):e18469. doi: 10.1111/jcmm.18469 (PMC11187809; doi:10.1111/jcmm.18469)

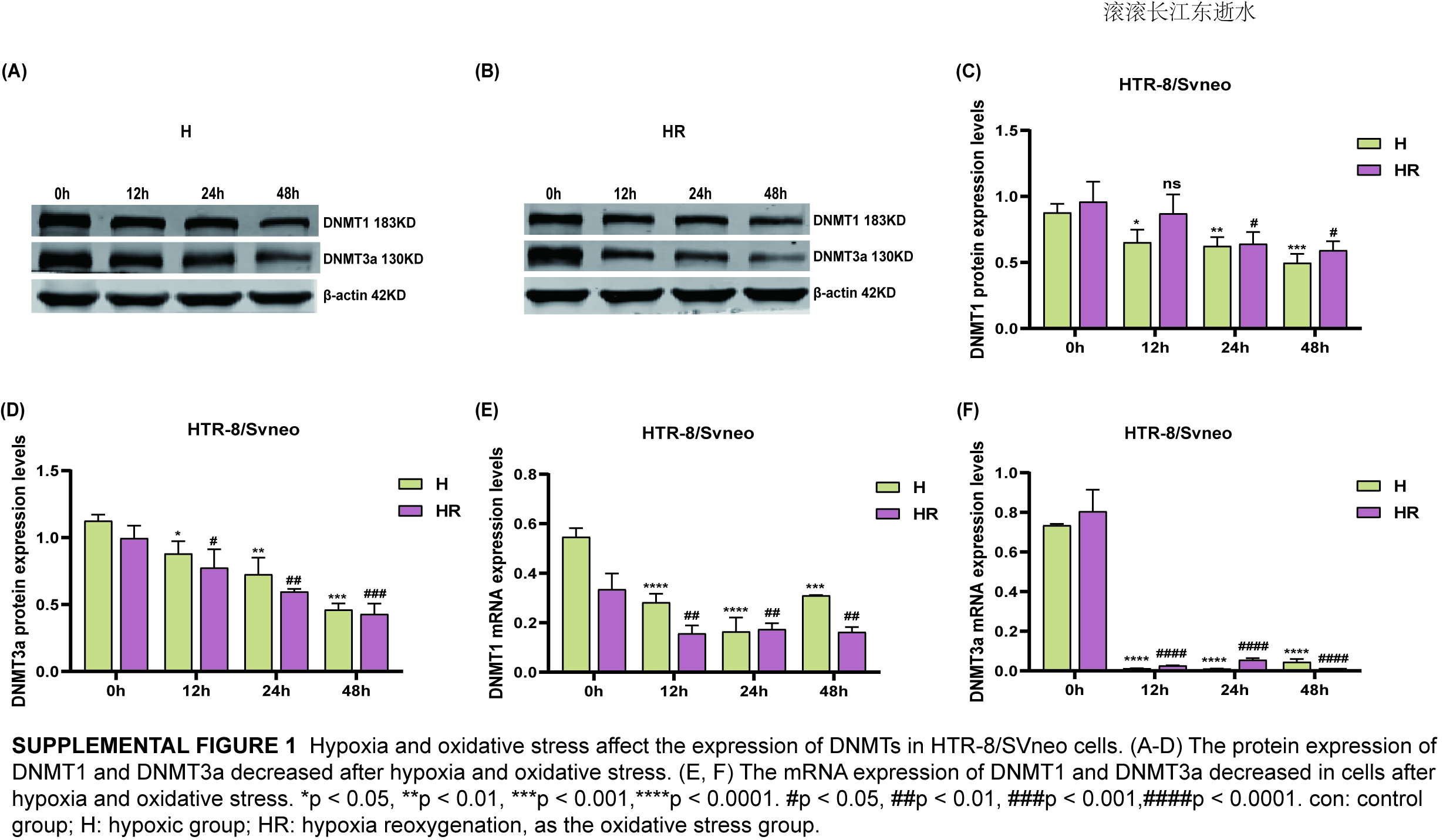

Supplement: Supplementary file 1 — Figure S1. [file JCMM-28-e18469-s001.tif]

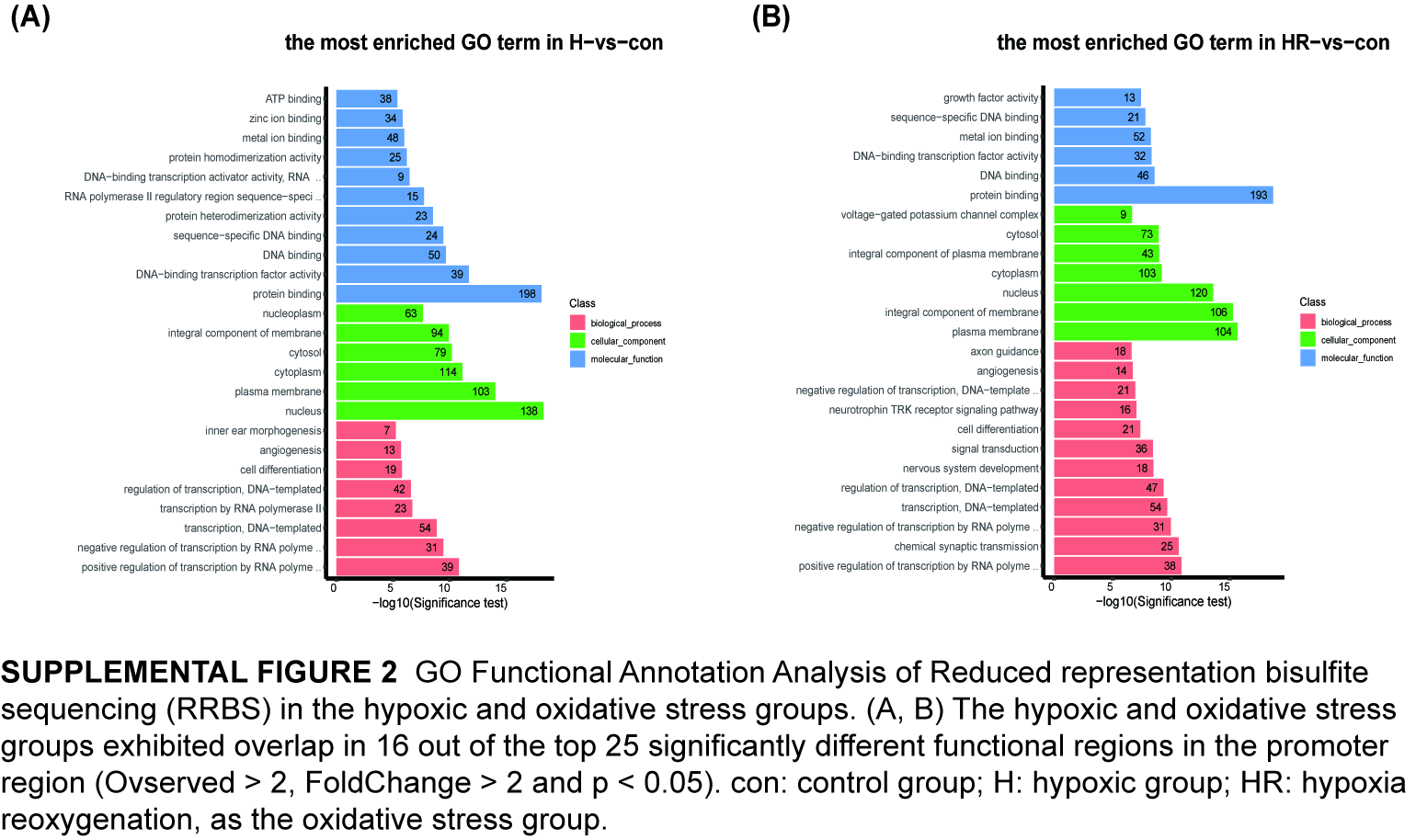

Supplement: Supplementary file 2 — Figure S2. [file JCMM-28-e18469-s002.tif]

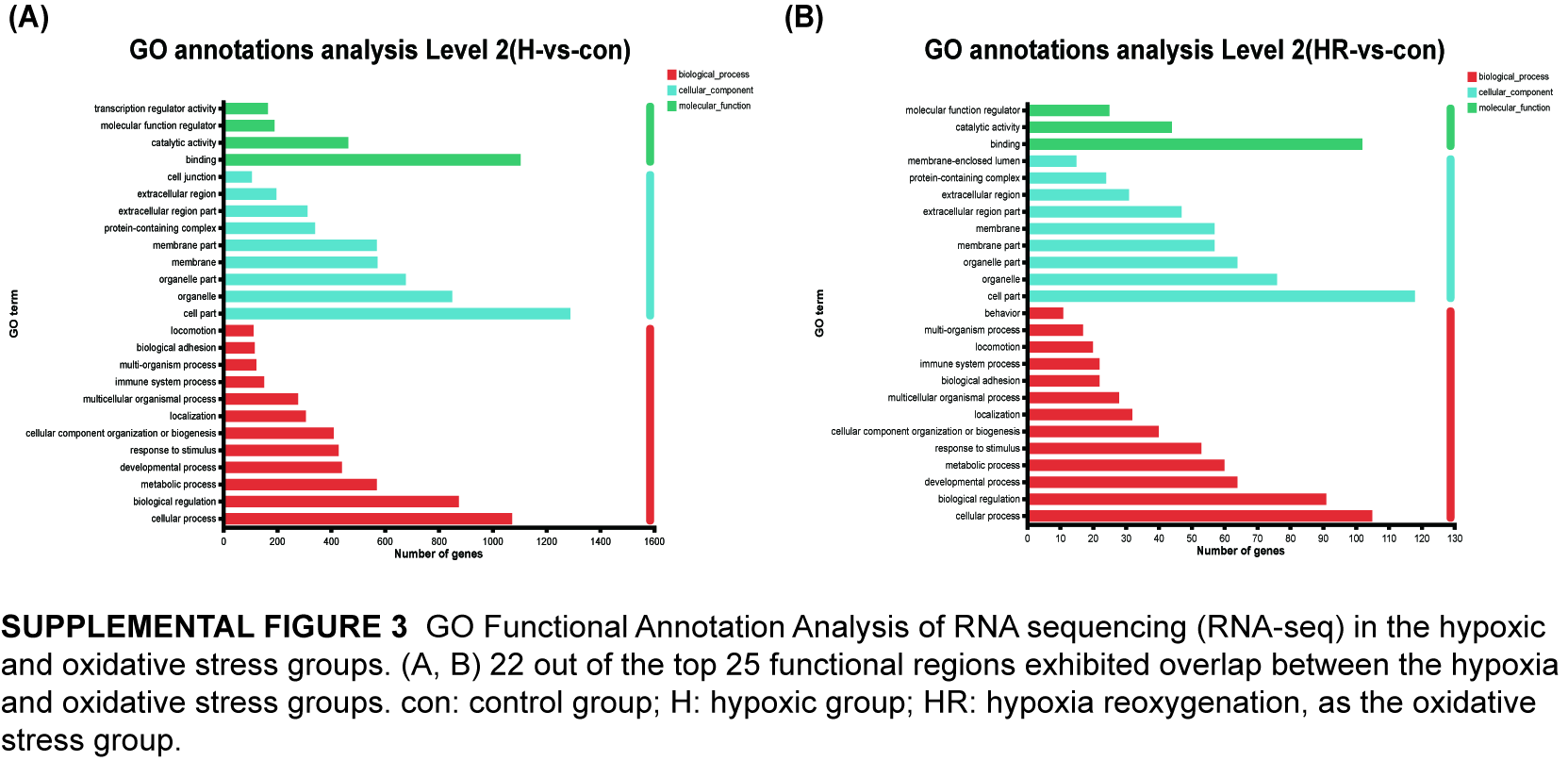

Supplement: Supplementary file 3 — Figure S3. [file JCMM-28-e18469-s003.tif]
